# A predictive model for lung cancer screening nonadherence in a community setting health-care network

**DOI:** 10.1093/jncics/pkad019

**Published:** 2023-04-07

**Authors:** Mehrad Bastani, Codruta Chiuzan, Gerard Silvestri, Suhail Raoof, Jesse Chusid, Michael Diefenbach, Stuart L Cohen

**Affiliations:** Department of Radiology, Northwell Health, Manhasset, NY, USA; Feinstein Institutes for Medical Research, Manhasset, NY, USA; Feinstein Institutes for Medical Research, Manhasset, NY, USA; Division of Pulmonary, Critical Care, Allergy and Sleep Medicine, Medical University of South Carolina, Charleston, SC, USA; Department of Pulmonary Medicine, Northwell Health, Donald and Barbara Zucker School of Medicine at Hofstra/Northwell, Northwell Health, Hempstead, NY, USA; Department of Radiology, Northwell Health, Manhasset, NY, USA; Department of Pulmonary Medicine, Northwell Health, Donald and Barbara Zucker School of Medicine at Hofstra/Northwell, Northwell Health, Hempstead, NY, USA; Feinstein Institutes for Medical Research, Manhasset, NY, USA; Department of Radiology, Northwell Health, Manhasset, NY, USA; Feinstein Institutes for Medical Research, Manhasset, NY, USA; Department of Pulmonary Medicine, Northwell Health, Donald and Barbara Zucker School of Medicine at Hofstra/Northwell, Northwell Health, Hempstead, NY, USA

## Abstract

**Background:**

Lung cancer screening (LCS) decreases lung cancer mortality. However, its benefit may be limited by nonadherence to screening. Although factors associated with LCS nonadherence have been identified, to the best of our knowledge, no predictive models have been developed to predict LCS nonadherence. The purpose of this study was to develop a predictive model leveraging a machine learning model to predict LCS nonadherence risk.

**Methods:**

A retrospective cohort of patients who enrolled in our LCS program between 2015 and 2018 was used to develop a model to predict the risk of nonadherence to annual LCS after the baseline examination. Clinical and demographic data were used to fit logistic regression, random forest, and gradient-boosting models that were internally validated on the basis of accuracy and area under the receiver operating curve.

**Results:**

A total of 1875 individuals with baseline LCS were included in the analysis, with 1264 (67.4%) as nonadherent. Nonadherence was defined on the basis of baseline chest computed tomography (CT) findings. Clinical and demographic predictors were used on the basis of availability and statistical significance. The gradient-boosting model had the highest area under the receiver operating curve (0.89, 95% confidence interval = 0.87 to 0.90), with a mean accuracy of 0.82. Referral specialty, insurance type, and baseline Lung CT Screening Reporting & Data System (LungRADS) score were the best predictors of nonadherence to LCS.

**Conclusions:**

We developed a machine learning model using readily available clinical and demographic data to predict LCS nonadherence with high accuracy and discrimination. After further prospective validation, this model can be used to identify patients for interventions to improve LCS adherence and decrease lung cancer burden.

Lung cancer (LC) remains the leading cause of cancer-related deaths nationwide, with an estimated 130 180 deaths in 2022 (1). The National Lung Screening Trial (NLST) found that annual low-dose computed tomography (LDCT) decreased mortality by 20% compared with chest radiography (2). Following the NLST, the United States Preventive Service Task Force (USPSTF) issued the first lung cancer screening (LCS) guideline in 2013, with a revision in 2021 to include a younger and less heavy smoking population (3,4). The current eligibility criteria (USPSTF 2021) include patients aged between 50 and 80 years with a smoking history of 20 pack-years who currently smoke or formerly smoked but quit within 15 years (4).

Favorable benefits of LCS programs in the NLST and the Dutch-Belgian Randomized Lung Cancer Screening Trial (Dutch acronym: NELSON study) trials were obtained because of the exceptionally high level of adherence (90%-95%) (5,6). Previously developed modeling work performed by a member of the Cancer Intervention and Surveillance Model Network found that an adherence rate of 46% would mitigate the mortality benefit of LCS by 50% (7). To achieve the full health benefits of LCS, successful guideline implementation, including returning for annual LCS (also known as adherence), is vital; however, there are many barriers to LCS adherence (8). One barrier to LCS in a community health-care setting is that some individuals might consider LCS as “a one-and-done” test, resulting in poor adherence to subsequent screens (9). Among other barriers, this mentality resulted in strikingly poor adherence to LCS ranging from 30 to 60 in real-world settings, reducing LCS effectiveness (9-11). Identification of patients at higher risk for LCS nonadherence is a critical first step toward improving adherence. Several studies have identified statistically significant clinical and demographic factors such as smoking status, screening site, race, and prior chest nodule findings associated with nonadherence to the first and second subsequent annual LCS (9-13). Although factors associated with LCS nonadherence have been identified, to the best of our knowledge, no models have been developed to predict LCS nonadherence risk on a patient level.

Predictive modeling is a statistical technique using machine learning (ML) and data mining to predict and forecast likely future outcomes with the aid of existing data (14). The purpose of this study is to develop a predictive model leveraging ML methodology that uses clinical and demographic factors associated with LCS adherence and readily available in the LCS registry to predict LCS nonadherence risk. Such a model can be used to identify patients for interventions to improve LCS adherence and decrease LC burden.

## Methods

### Study participants

Patients included in this study were enrolled in our LCS program from January 1, 2015, to January 1, 2018, who fulfilled the 2013 USPSTF LCS guidelines (aged 55-80 years, smoking a minimum 30 pack-years, and within 15 years for former smokers). Those who died during or before their subsequent screen or short-term follow-up were excluded. This study was conducted in a community, multi-integrated, health-care network setting.

For data source, we used the electronic medical records, radiology information system, and Institutional American College of Radiology LCS database to extract clinical and demographic variables. The study was performed with institutional review board approval and waiver of informed consent, and the data underlying this article cannot be shared publicly. The data will be shared on reasonable request to the corresponding author.

### LCS program

Ten LCS imaging sites associated with our health system are grouped into 3 geographic regions with an assigned advanced practice provider (APP) for each region. Once a referral is generated by a provider within our health system, patients are contacted via phone calls by an APP to obtain information and confirm eligibility for an office visit. The shared decision-making visit may be carried out by the APP or the primary provider and is left at the discretion of the provider.

Shared decision making in the context of LCS entails a discussion about the potential benefit of decreasing the risk of LC mortality as well as the potential harms, including false-positive results, complications because of positive screens, overdiagnosis, and radiation exposure (15). Patients may also call a dedicated hotline and be assessed for their eligibility for LCS. After the office visit and LDCT for the LCS, patients with negative findings are scheduled for an annual screen and will receive 2 reminder letters before their annual subsequent LCS due date.

### Study variables

#### Outcome

The target variable was defined as adherence to LCS with subsequent computed tomography (CT) after baseline examination specific to LungRADS findings and follow-up recommendations. Adherence to LCS for LungRADS 0-2 (negative findings) was defined as returning within 11-15 months after baseline examination, as defined by NLST, for the second screen (2). A time interval of ±3 months was used to define adherence for LungRADS 3 and 4A, patients representing short-term follow-up between 3 and 9 months after baseline screening for LungRADS 3 and 0 and 6 months after baseline LCS for LungRADS 4A. Adherence for patients with LungRADS 4B/X was defined as returning for the short-term follow-up to 3 months after baseline screening (0-3 months after LCS). It is worthwhile to note that non-LCS chest CTs were not used for adherence to screening.

#### Predictors

Patients’ demographic and clinical variables based on prior studies were chosen as predictors because they are clinically relevant and readily available in most LCS registries from the baseline LCS exam. Demographic variables include sex, age, race, screening sites, insurance, and median household income. Clinical variables include prior chest LCS findings (LungRADS score), smoking history and status, and referral specialty. All variables were treated as categorical variables. Age was categorized into 4 groups (55-59 years, 60-69 years, 70 years, and older than 70 years). Smoking status was defined as current or former smoker. Race was categorized into 4 groups: White, African American, Asian, and not reported. LungRADS scores were considered as 1, 2, 3, 4A, 4B, and 4X on the basis of the most recent guidelines (16). Site was categorized into 3 LCS regions (R1-R3) for each of the 10 sites according to geographical locations and operations structure. Each one of these regions was administered by an assigned APP. Our system reflects the spectrum of LCS programs carried out at tertiary referral centers, community hospitals, health clinics, and private physicians’ offices. However, the rate of patients is not equally distributed among regions (R1-R3), and it is dependent on the number of suspicious LC patients in the area. Individuals’ median household incomes were categorized into 3 groups according to the 50th (median) and 75th percentile of the household income distribution of their associated zip codes. Seventeen provider specialties were categorized into 5 groups: internal or family medicine, pulmonary diseases, thoracic surgery, physician assistant or nurse practitioner, and others. The designation of the referring provider was based on the person ordering the baseline exam using an online National Provider Identifier lookup. Insurance types of patients were grouped into 4 main categories: Medicare, Medicaid, private, and others. All nonreported values in the predictor variables were coded as a separate category named “unknown” instead of a complete case analysis. The tree-based methods used in this article are capable of considering the “unknown” category as a predictor and show their statistical significance in the outcomes.

### Predictive model development and analysis

We used parametric and nonparametric models for the predictive modeling of LCS nonadherence. First, univariate regression analyses are performed to detect the statistically significant factors associated with nonadherence to the first subsequent LCS. Then, we fit a logistic regression among parametric models with backward and stepwise elimination using Akaike information criterion (AIC) for selecting the variables that will provide the best fit for the model. The AIC is an estimator of prediction error, and thereby the relative quality of statistical models for a given set of data in which a lower AIC value indicates a better-fit model (17). We further applied random forest and gradient boosting (XGBoost) as tree-based methods among nonparametric algorithms. Random forest is the variation of a decision tree with the difference that it uses the concept of bagging to generalize the model (18). It involves generating N bootstrapped samples, which are trained on a different set of predictors in the dataset (ie, age, smoking status, etc, in the LCS database), which in effect leads to N different strong decision trees. These N different trees are then aggregated into a single model for generalizability purposes (19). Gradient boosting is another variation of ensemble learning using independent models (20). For all of the predictive models applied in this study, 10-fold cross-validation and bootstrapping methods are applied to quantify the uncertainty associated with a given estimator or statistical learning method. The cross-validation and bootstrapping methods estimate the standard errors of the coefficients from predictive methods.

The performance and discriminative ability of all 3 models were assessed using the area under the ROC curve (AUC) on the basis of 1000 bootstrap samples. The model with the highest AUC was used to describe the variable importance of baseline features. All statistical analyses were performed in R (v. 4.1.0) (21) using a 2-sided type I error of 0.05.

## Results

**Figure 1.**
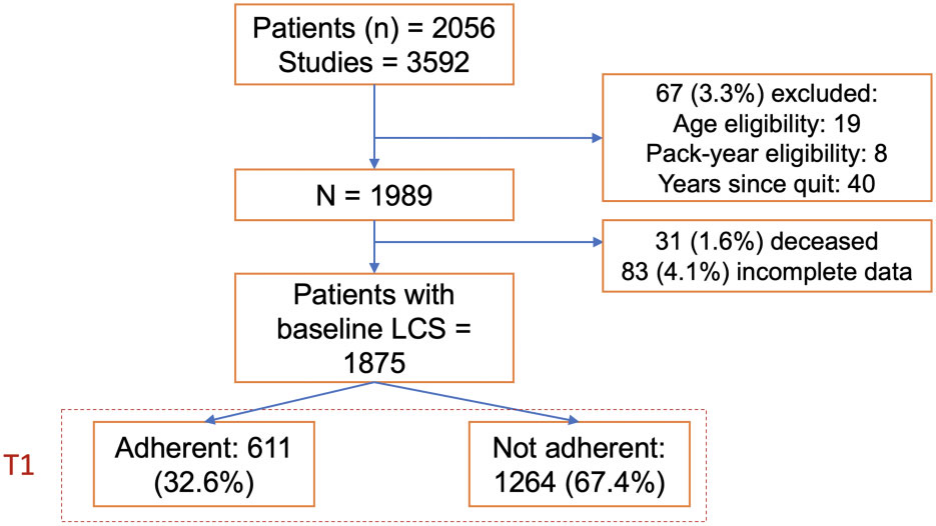
Flow diagram of patient inclusion in the study cohort. LCS = lung cancer screening.

A total of 2056 individuals from January 1, 2015, to January 1, 2018, who received LCS were identified from the institutional LC registry. Figure 1 presents the flow diagram for patient inclusion in this study. Of the 2056 patients with LCS, 67 (3.3%) were excluded because of age and smoking history ineligibility according to USPSTF guidelines. Of 1989 remaining patients, 114 further individuals were excluded because they were deceased (n_deceased_ = 31) or because of the incomplete radiology data for the subsequent screening (n_incomplete_ = 83). The final dataset included N = 1875 observations of baseline LCS, with 1264 nonadherent patients (67.4%) for the first subsequent annual LCS.

Table 1 outlines the demographic and clinical characteristics of the study cohort stratified by first subsequent adherence status. Comparative analysis of patients’ characteristics (univariate regression) revealed that sex, race, smoking status, baseline screening findings (LungRADS score), referral specialty, and screening sites were among the statistically significant factors associated with nonadherence to the first subsequent LCS. It is worth noting that 104 (5.5%) and 21 (1.1%) of race and smoking status information from the final cohort of patients (N = 1875) were not available, respectively. We did not want to exclude all the patient data from the model development because of the missing race and smoking variables (2 of 9 clinical and demographic variables), so these variables were categorized as “unknown” in Table 1. Furthermore, all the predictive models proposed in this article are well capable of treating missing values by considering them as a separate category (ie, “unknown”) to show their statistical significance in the outcomes. The results of all 3 predictive methodologies are summarized in Table 2, setting nonadherence as a positive class. Both tree-based methods (random forest and gradient boosting) outperformed the logistic regression method when the AUC, the area under the precision-recall curve, positive predictive value (PPV), negative predictive value, sensitivity, specificity, and accuracy were considered as measurements. It is worth noting that both logistic regression with all the predictors and parsimonious logistic regression with statistically significant features based on univariate regression resulted in the same results because of the ability of regularization to handle overfitting.

**Table 1.** Patient characteristics at baseline screening (N = 1875)^a^

Characteristics	Nonadherent (N_1_ = 1264)	Adherent (N_2_ = 611)	*P* (univariate regression)
Sex			.043
Female	548 (43%)	297 (49%)	
Male	716 (57%)	314 (51%)	
Age, y			.592
Mean (SD)	63.8 (5.7)	64.1 (5.6)	
Median (IQR)	63.1 (59.2, 67.9)	63.3 (59.4, 68.5)	
Age categories			.867
55-59	383 (30.3%)	179 (29.2%)	
60-69	662 (52.4%)	321 (52.7%)	
≥70	219 (17.4%)	111 (18.1%)	
Race			.044
African American	113 (8.9%)	57 (9.3%)	
Asian	64 (5.1%)	36 (5.9%)	
Unknown	83 (6.5%)	21 (3.4%)	
White	1004 (79.5%)	497 (81.4%)	
LungRADS score at baseline screen			<.001
1	393 (31.1%)	138 (22.5%)	
2	758 (59.9%)	323 (52.7%)	
3	75 (5.9%)	77 (12.6%)	
4A	25 (2.0%)	47 (7.7%)	
4B	7 (0.6%)	16 (2.6%)	
4X	6 (0.5%)	10 (1.6%)	
Smoking status			<.001
Currently smoke	690 (54.6%)	308 (49.1%)	
Formerly smoked	556 (43.9%)	300 (50.2%)	
Unknown	18 (1.5%)	3 (0.6%)	
Site			.015
R1	62 (4.9%)	90 (14.7%)	
R2	694 (54.8%)	300 (49.3%)	
R3	508 (40.3%)	221 (36.0%)	
Median household income			.101
Mean (SD)	86 050 (25 912)	83 470 (25 750)	
Median (IQR)	87 327 (68 890-100 250)	85 672 (67 914-101 434)	
Categories			.243
<85 k/y	599 (47%)	305 (49.9%)	
85-100 k/y	321 (25%)	163 (26.7%)	
>100 k/y	344 (27%)	143 (23.4%)	
Specialty			<.001
Internal/family medicine	647 (51.1%)	234 (38.3%)	
Other	73 (5.8%)	21 (3.6%)	
Physician assistant/nurse practitioner	36 (2.8%)	37 (6.0%)	
Pulmonary	437 (34.6%)	278 (45.3%)	
Thoracic	71 (5.61%)	41 (6.7%)	
Insurance			.302
Medicaid	159 (13%)	59 (9.6%)	
Medicare	517 (41%)	260 (43%)	
Other	73 (5.8%)	33 (5.5%)	
Private	515 (41%)	259 (42%)	

a IQR = interquartile range.

**Table 2.** Results of machine learning models to predict nonadherence to LCS^a^

Models	AUC (95% CI)	PPV (95% CI)	NPV (95% CI)	Sensitivity (95% CI)	Specificity (95% CI)	Accuracy (95% CI)
Parsimonious logistic regression	0.68 (0.65 to 0.70)	0.63 (0.57 to 0.68)	0.73 (0.70 to 0.75)	0.92 (0.90 to 0.93)	0.28 (0.25 to 0.32)	0.71 (0.69 to 0.73)
Random forest	0.78 (0.76 to 0.80)	0.95 (0.92 to 0.97)	0.83 (0.81 to 0.84)	0.98 (0.98 to 0.99)	0.57 (0.53 to 0.61)	0.85 (0.83 to 0.86)
Gradient boosting	0.89 (0.87 to 0.90)	0.83 (0.79 to 0.86)	0.82 (0.80 to 0.84)	0.94 (0.93 to 0.95)	0.58 (0.54 to 0.63)	0.82 (0.81 to 0.84)

a AUC = area under the receiver operating characteristic (ROC) curve; CI = confidence interval; LCS = lung cancer screening; NPV = negative predictive value; PPV = positive predictive value.

Random forest yielded comparable or even higher PPV (0.95 vs 0.83), negative predictive value (0.83 vs 0.82), sensitivity (0.98 vs 0.94), specificity (0.57 vs 0.58), and accuracy (0.85 vs 0.82) compared with gradient boosting methodology. However, gradient boosting resulted in higher AUC, which indicates a very good ability to discriminate patients at risk of being nonadherent.

**Figure 2.**
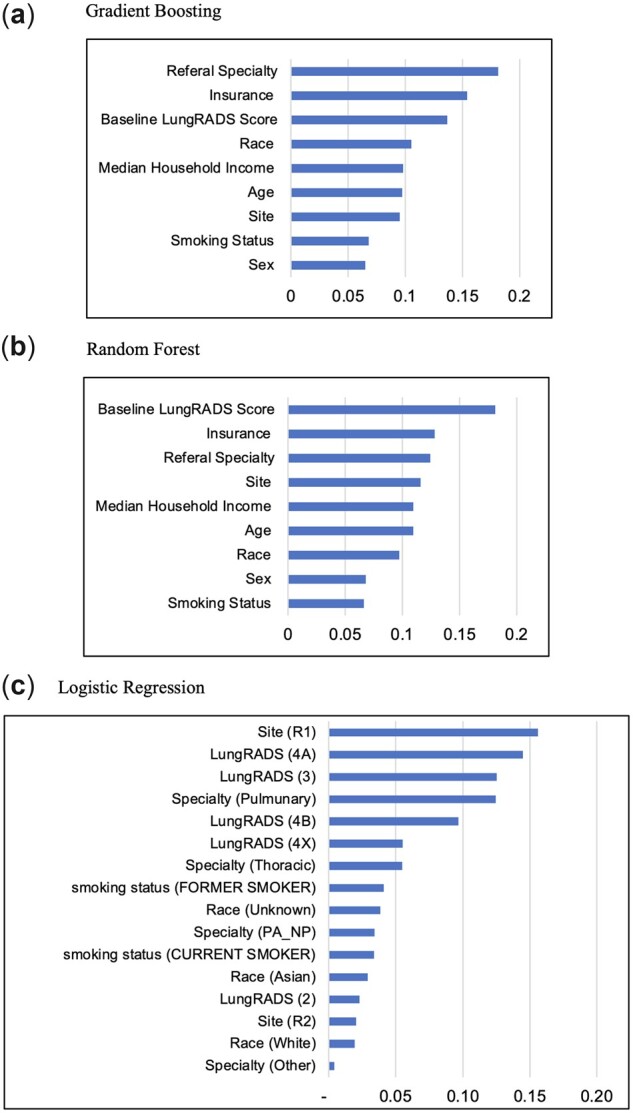
Relative importance of top predictive variables identified by **A**) gradient boosting, **B**) random forest, and **C**) logistic regression.

Figure 2 represents the relative importance of predictors that the gradient-boosting method used to derive the nonadherence prediction to the first subsequent LCS compared with the random forest and logistic regression methods. Referral specialty, insurance type, baseline LungRADS score, and race were among the top 4 predictors used by the gradient-boosting algorithm to yield the best AUC and area under the precision-recall curve. In the same vein, random forest methodology used baseline LungRADS score, insurance, referral specialty, and site to derive its prediction. Variable importance of logistic regression is only accessible on the basis of factor levels yielding screening site, LungRADS score, and referral specialty among the top predictors of nonadherence to first subsequent LCS.

## Discussion

We developed and internally validated a multivariable model for predicting nonadherence to LDCT for LCS. Although several prior studies have identified the factors associated with adherence to LCS, our study is, to our knowledge, the first to provide a risk predictive model for nonadherence to LCS. Among the predictive modeling methods, gradient boosting outperformed random forest and logistic regression when AUC was used as a measurement, showing a promising ability in discriminating patients at risk of being nonadherent after a baseline LCS. Three of the predictors with the highest impact on nonadherence to LCS in the gradient-boosting model were referral specialty, insurance type, and baseline nodule findings (LungRADS scores). Referral specialty and baseline nodule findings were also among the factors associated with adherence to LCS among patients with baseline positive findings in prior studies (9-11). Calculating the risk of nonadherence to LCS through this automated and systematic approach can help to prospectively identify individuals who are at high risk for nonadherence and increase the health benefits of screening.

This predictive model expands on the previous studies in the literature on the low adherence of LCS in community health-care settings. LCS adherence in the general population is low (30%-60%) and even lower among uninsured patients (22), reducing LCS effectiveness (10,11). Across our network that serves a diverse patient population, we observe adherence rates for the first subsequent exam after baseline screening at approximately 33%. This adherence rate is strikingly low compared with that of other screening programs, such as breast and cervical cancer screening (22-24). Similar low adherence rates to LCS have been consistently observed in community health-care settings (9-11,25,26). Interventions to improve LCS adherence include frequent reminders for follow-up and direct access to providers to discuss screening as well as electronic methods of communication (27-29). However, patients in need of these interventions must be identified so clinical resources can be efficiently deployed. The purpose of our predictive model was to develop a personalized predictive risk model for patient nonadherence to subsequent LCS to identify individuals in need of targeted outreach to improve adherence. This study is innovative because it is the first study, to our knowledge, to use computational modeling to predict the risk of patient nonadherence to LCS to maximize the effectiveness of LCS.

Our predictive model’s high AUC, high sensitivity (when nonadherence is set as a positive class), and high PPV make it well suited to help cancer screening sites more effectively deploy resources to improve LCS adherence. For example, “high-risk” patients with a higher probability of nonadherence, as identified by the model, could be assigned to navigators or APPs, who have been shown to statistically significantly improve cancer screening adherence, for targeted outreach (6). Because the availability of navigators and APPs is limited by the capacity of the cancer screening center, a risk predictive model could help them as a supportive tool to contact patients with a higher risk of nonadherence. Our model ranks the patients based on their nonadherence risk, which enables the providers to use their time more efficiently by prioritizing patients with a higher risk of nonadherence. The lack of a feedback loop with the health-care system after the LCS might contribute to the challenges in engaging the patient in screening adherence. Although our proposed predictive model can potentially find the patient at a higher risk of nonadherence, a consistent loop for inpatients and outpatients is required to increase adherence and early diagnosis and ultimately decrease mortality.

The top 3 predictors used by our model to predict the nonadherence rates are referral specialty, insurance types, and baseline LungRADS findings. All these predictors were among the factors associated with adherence to LCS in prior studies, and our study shows the performance when they were used for predicting the upcoming LCS individuals. Our results showed that patients diagnosed with suspicious baseline nodule findings (LungRADS 3 and 4A/B/X), with Medicare and private insurance, and referred by pulmonary specialists were at a lower risk of being nonadherent. These results demonstrate the potential benefit of incorporating a risk predictive model for targeted reminders (ie, emails) among patients at a higher risk of nonadherence to increase adherence (5).

Both tree-based models (random forests and gradient boosting) applied in this study demonstrated much higher performance compared with the logistic regression for the following potential reasons. First, logistic regression assumes that the data are linearly separable; however, tree-based models do not require the linear separation of the initial data. Second, the majority of the data used to build our predictive model are categorical. Tree-based models are known to outperform logistic regression with categorical data. Finally, tree-based models can automatically consider interactions and correlations between variables that could exist in our dataset.

Our predictive model demonstrates promising results in the detection of patients at higher risk of nonadherence to LCS. However, the generalizability of the model needs to be assessed in future studies. External validation and refinement of the developed predictive model with larger datasets can shed light on using ML-based models in LCS practice.

### Limitations

Our study had several limitations. First, our sample was limited to a single health-care network in New York, and it may not represent national demographics. Second, there is no SD of adherence to LCS, which could cause the seemingly wide variation of adherence to LCS in previous studies; however, we used the NLST definition of adherence to LCS to make our results analogous. Third, some patients may have undergone chest CT for other reasons and were not recorded in our LC registry. Fourth, our model is based on the information of patients who started their LCS with our health-care network but may have continued their screening in other centers. Finally, our predictive model needs to be externally validated using a national dataset for generalizability purposes and to avoid overfitting.

Adherence to LCS is strikingly low nationwide, and we have developed a predictive model that can improve screening adherence by targeting patients at a higher risk of not returning for the subsequent screen. Future work should focus on expanding the LCS dataset to include a larger number of patients from multiple health networks to externally validate and refine the predictive model.

## Data Availability

The study was performed with institutional review board approval and waiver of informed consent, and the data underlying this article cannot be shared publicly due to the privacy of individuals who participated in the study. Aggregated summaries without individual data were shared in the manuscript.
